# Editorial: Influence of lifestyle factors in the management of diabetes mellitus

**DOI:** 10.3389/fendo.2023.1258766

**Published:** 2023-08-10

**Authors:** Celestino Sardu, Gaetano Santulli, Nunzia D’ Onofrio

**Affiliations:** ^1^ Department of Advanced Medical and Surgical Sciences, University of Campania, “Luigi Vanvitelli”, Naples, Italy; ^2^ Department of Medicine, Fleischer Institute for Diabetes and Metabolism (FIDAM), Einstein-Mount Sinai Diabetes Research Center (ES-DRC), Albert Einstein College of Medicine, New York, NY, United States; ^3^ Department of Advanced Biomedical Science, “Federico II” University, Naples, Italy; ^4^ Department of Biochemistry, Biophysics and General Pathology, School of Medicine and Surgery, University of Campania, Naples, Italy

**Keywords:** diabetes mellitus, inflammation, oxidative stress, drug therapy, lifestyle

Diabetes mellitus (DM) is a multi-factorial metabolic disease that affects approximately 462 million individuals, corresponding to 6.28% of the world’s population (4.4% of those aged 15–49 years, 15% of those aged 50–69, and 22% of those aged 70+), with a prevalence rate of 6,059 cases per 100,000 (1). DM accounts for over 1 million deaths per year, making it the ninth leading cause of mortality (1). The DM burden is rising globally and at a much faster rate in developed regions, such as Western Europe, with equal gender distribution, and the incidence peaks at approximately 55 years of age (1).

In this scenario, we expect a global prevalence of DM of approximately 7,079 individuals per 100,000 by 2030 (1). To date, the rising burden of DM is a major concern in healthcare worldwide, and there is a need for urgent public health and clinical preventive measures (1). In non-Western countries, in a cohort of 215,041 elderly adults living in China (102,692 men and 112,349 women), authors reported a prevalence of self-reported diabetes of approximately 8.7%, with the highest prevalence in Beijing (20.8%) and the lowest prevalence in Xizang (0.9%), (Hu et al.). Notably, urban areas, older age, female, higher income, poor sleep quality, and some other factors were potential risk factors for diabetes (Hu et al.). Conversely, sociodemographic and behavioral factors could be linked to the low awareness of DM medication (Khoiry et al.). Indeed, irregular blood glucose monitoring without any comorbidity, never having any general medical checkup, 26–35 years of age, 36–45 years of age, and having no health insurance coverage were significantly associated with low awareness of diabetes medication (Khoiry et al.).

Notably, the complexity of oral anti-diabetic drug (OAD) regimens could affect the quality of life (QOL) and treatment satisfaction (Chang et al.). Indeed, a study conducted in Taiwan showed a significantly greater effect on QOL among patients with fewer OAD classes and higher treatment satisfaction (Chang et al.). Parallelly, new anti-DM treatments could effectively ameliorate the glucose and blood lipid metabolism via effects on the intestinal flora in type 2 DM (T2DM) patients (Peng, X. et al.). In this context, the *Cyclocarya paliurus* leaves’ extracts (CP) display a more beneficial effect in the alleviation of T2DM-associated metabolic phenotypes than glipizide by regulating gut microbiota and metabolites in T2DM patients, with no significant effects on liver and kidney function (Peng, X. et al.). The liver activity could itself play a relevant role in the metabolic abnormalities of DM by controlling lipid and glucose homeostasis and feeding metabolites (Chen et al.). Indeed, authors observed that feeding could induce the release of hepatokines, which regulates glucose and lipid metabolism, and these feeding-induced hepatokines act on multiple organs to regulate glucolipotoxicity and thus influence the development of T2DM (Chen et al.).

Regarding the negative role played by lipid metabolism in DM patients, authors found that clonal hematopoiesis could be evaluated as a novel risk factor for T2DM patients with hypercholesterolemia (Kim et al.). Indeed, the clonal hematopoiesis of indeterminate potential (CHIP) is associated with atherosclerosis, cardiovascular disease (CVD), and new-onset T2DM (Kim et al.). Notably, the subjects with CHIP and hyper-LDL-cholesterolemia had approximately twice the risk of diabetes than subjects without CHIP and with low LDL cholesterol (Kim et al.). Thus, there could be a synergism between CHIP and high LDL cholesterol as a high-risk factor for diabetes (Kim et al.).

In this context, it is well known that DM causes an increase in inflammatory and oxidative stress, negatively affecting glucose homeostasis and insulin resistance and worsening clinical outcomes (2, 3). Indeed, over-inflammation/oxidative stress is a leading cause of atherosclerosis, atherosclerotic plaque fissuration, and CVDs in DM patients (2, 3), such as in the overall population (4, 5) and in gender-specific cohorts of patients (Wu et al.). Moreover, abnormalities of oxidative balance score (OBS) and high OBS are negatively associated with diabetes risk in a gender-dependent manner (Wu et al.).

Conversely, personality factors and health status influence resilience, and coping strategies could influence diabetic subjects (Rivera-Picón et al.). Indeed, concerning health status, the absence of pathology is related to using rational strategies more than to diagnosing diabetes (Rivera-Picón et al.). In the clinical setting, although diabetes care is improving, there are still cases that are poorly managed with adverse clinical outcomes (2–4). Indeed, DM is a leading cause of CVDs, and hospitalizations and deaths in developed countries (5). Conversely, DM could negatively affect other clinical conditions, such as cognitive decline (Wang et al.). Indeed, people with DM described misconceptions about their cognitive decline and suffered from them during disease management (Wang et al.). Intriguingly, authors found that poor glycemic control could impair the brain networks responsible for learning, memory, and controlled reactivity to food in adolescents with type 1 diabetes whose glycemic control is poor (Litmanovitch et al.). Thus, we need to support disease management with cognitive decline in clinical practice (Wang et al.), and improve glycemic control to ameliorate brain functions (Litmanovitch et al.).

However, from here, we could say that the DM prevention necessitates an integrated and holistic strategy to ameliorate glycemic control based on the condition’s cause. Indeed, inadequate glycemic management impacts the usage of healthcare resources, medical expenses, and death rates dramatically. Furthermore, in the current topic, we focused, on one side, on the prevention of DM (lifestyle modification) and its complications (best glycemic control and anti-diabetic medications) and, on the other side, on the early treatment of CVDs linked to DM (drug and interventional treatments). As an example, regarding lifestyle modification, we could promote weight loss and physical activity (Brinkmann et al.). Indeed, obesity is a major risk factor for DM, which is, in turn, a significant risk factor for CVDs such as coronary artery disease and stroke (Chung et al.). Thus, in a study population of 24,346 participants, of whom 8,334 (mean age, 50.6 ± 11.0 years) were male and 16,012 (mean age, 50.5 ± 10.1 years) were female, authors found strong associations between the studied obesity-related indices and incidence of DM, and sex differences (Chung et al.). Hence, to better control DM, reducing body weight may be beneficial in addition to lifestyle modifications, diet control, and pharmacological interventions (Chung et al.).

Conversely, after a certain period of low-volume high-intensity interval training (LVHIIT), glycemic control, insulin resistance, body weight, lipid profile, and cardiorespiratory outcomes were significantly improved in T2DM patients (Peng, Y. et al.). This concept has been applied also to a cohort of 4,196 German company employees and divided into three risk groups based on their European Society of Cardiology–Systematic Coronary Risk Evaluation score (ESC-SCORE), (Brinkmann et al.). In these subjects, authors found that the ESC-SCORE changes from baseline differed significantly between the groups, with the intervention group achieving more favorable results in all follow-up visits 6, 12, 24, and 36 months later (at each time point: ITT: *p* < 0.001; PP: *p* ≤ 0.010) (Brinkmann et al.). Thus, they found the feasibility of attracting employees with pre-DM/DM at high cardiovascular (CVD) mortality risk to participate in a multimodal lifestyle program following a free CVD mortality risk screening at their workplace (Brinkmann et al.). The lifestyle intervention used in the PreFord study shows high potential for improving the health of company employees with pre-DM/DM in the long term (Brinkmann et al.). In the lifestyle intervention, we could report the laughter yoga and its effects on glycemic control among individuals with T2DM (Hirosaki et al.). The proposed study intervention consisted of a 12-week laughter yoga program that resulted in (in the laughter yoga group) a significant improvement in HbA1c levels and an increase in sleep duration (Hirosaki et al.). However, having fun could be a self-care intervention to ameliorate DM status (Hirosaki et al.).

According to the published articles on the current Research Topic, we could report that lifestyle factors could negatively influence and condition the management and clinical outcomes of DM. The more robust control of lifestyle factors could, on one side, result in the amelioration of glucose homeostasis and insulin resistance in DM patients. This could be seen as a glucose-dependent effect. On the other side, stronger control of lifestyle factors could reduce inflammatory/oxidative stress in DM patients (Wu et al.). This could be evidenced by the glucose-independent effect of lifestyle factor control. Finally, the control of lifestyle factors could reduce CVD disease and mortality via glucose-dependent and -independent effects. Furthermore, we might promote the control of lifestyle factors as a relevant therapeutic strategy for managing and treating patients living with DM.

## Author contributions

CS: Conceptualization, Investigation, Writing – original draft, Writing – review & editing. GS: Writing – review & editing, Investigation. ND’O: Investigation, Writing – review & editing.
